# Proarrhythmia in the p.Met207Val PITX2c-Linked Familial Atrial Fibrillation-Insights From Modeling

**DOI:** 10.3389/fphys.2019.01314

**Published:** 2019-10-22

**Authors:** Jieyun Bai, Yaosheng Lu, Andy Lo, Jichao Zhao, Henggui Zhang

**Affiliations:** ^1^Department of Electronic Engineering, College of Information Science and Technology, Jinan University, Guangzhou, China; ^2^Auckland Bioengineering Institute, The University of Auckland, Auckland, New Zealand; ^3^Biological Physics Group, School of Physics & Astronomy, University of Manchester, Manchester, United Kingdom; ^4^Pilot National Laboratory for Marine Science and Technology, Qingdao, China

**Keywords:** atrial fibrillation, PITX2c, modeling and simulation, human atrial action potential model, electrical and structural remodeling, gene regulation, transcription factors, single nucleotide polymorphism

## Abstract

Functional analysis has shown that the p.Met207Val mutation was linked to atrial fibrillation and caused an increase in transactivation activity of PITX2c, which caused changes in mRNA synthesis related to ionic channels and intercellular electrical coupling. We assumed that these changes were quantitatively translated to the functional level. This study aimed to investigate the potential impact of the PITX2c p.Met207Val mutation on atrial electrical activity through multiscale computational models. The well-known Courtemanche-Ramirez-Nattel (CRN) model of human atrial cell action potentials (APs) was modified to incorporate experimental data on the expected p.Met207Val mutation-induced changes in ionic channel currents (*I*_*NaL*_, *I*_*Ks*_, and *I*_*Kr*_) and intercellular electrical coupling. The cell models for wild-type (WT), heterozygous (Mutant/Wild type, MT/WT), and homozygous (Mutant, MT) PITX2c cases were incorporated into homogeneous multicellular 1D and 2D tissue models. Effects of this mutation-induced remodeling were quantified as changes in AP profile, AP duration (APD) restitution, conduction velocity (CV) restitution and wavelength (WL). Temporal and spatial vulnerabilities of atrial tissue to the genesis of reentry were computed. Dynamic behaviors of re-entrant excitation waves (Life span, tip trajectory and dominant frequency) in a homogeneous 2D tissue model were characterized. Our results suggest that the PITX2c p.Met207Val mutation abbreviated atrial APD and flattened APD restitution curves. It reduced atrial CV and WL that facilitated the conduction of high rate atrial excitation waves. It increased the tissue's temporal vulnerability by increasing the vulnerable window for initiating reentry and increased the tissue spatial vulnerability by reducing the substrate size necessary to sustain reentry. In the 2D models, the mutation also stabilized and accelerated re-entrant excitation waves, leading to rapid and sustained reentry. In conclusion, electrical and structural remodeling arising from the PITX2c p.Met207Val mutation may increase atrial susceptibility to arrhythmia due to shortened APD, reduced CV and increased tissue vulnerability, which, in combination, facilitate initiation and maintenance of re-entrant excitation waves.

## Introduction

The most common arrhythmia atrial fibrillation (AF) increases with age and is associated with adverse events (such as heart failure, stroke, hypertension and diabetes) (Heijman et al., 2018). These cardiac disorders are thought to promote AF which is characterized by uncoordinated patterns of atrial electrical activation and a fast and irregular heartbeat (Hansen et al., 2015). Whilst the precise mechanisms underlying AF are complex and poorly understood, AF-induced ionic remodeling and structural cardiac diseases are major factors in initiating and sustaining AF (Grandi et al., 2011; Colman et al., 2013; Koivumäki et al., 2014). However, genome-wide association studies suggested genetic variation contributes to AF susceptibility, with >100 AF-associated loci reported to date (Nielsen et al., 2018), including the atrial-selective transcription factor PITX2 (paired like homeodomain-2) that regulates membrane effector genes associated with AF (Gudbjartsson et al., 2007; Chinchilla et al., 2011; Kirchhof et al., 2011; Qiu et al., 2014; Tao et al., 2014; Lozano-Velasco et al., 2015; Pérez-Hernández et al., 2015; Bai et al., 2018; Mechakra et al., 2019). In these studies, Mechakra et al. (2019) identified a non-synonymous mutation c.619A>G (p.Met207Val, rs138163892) of PITX2. Functional analysis of the transactivation activity of wild-type and variant PITX2c revealed a gain-of-function of PITX2c (the PITX2 c isoform), leading to an increase in the mRNA level of KCNH2 (the α subunit of *I*_*Kr*_), KCNQ1 (the α subunit of *I*_*Ks*_), SCN1B (the β1 subunit of sodium channels that modulates *I*_*NaL*_), GJA5 (Cx40), and GJA1 (Cx43) (Mechakra et al., 2019).

*I*_*Kr*_, *I*_*Ks*_, and *I*_*NaL*_ regulate late repolarization of action potentials (APs), and Cx40 and Cx43 mediate intercellular electrical coupling via gap junctions (Dhillon et al., 2014). Ionic remodeling due to changes in potassium currents (Caballero et al., 2010; González de la Fuente et al., 2012; Pérez-Hernández et al., 2015) and *I*_*NaL*_ (Sossalla et al., 2010), and structural remodeling arising from abnormalities in Cx40 and Cx43 (Polontchouk et al., 2001; Nao et al., 2003; Wetzel et al., 2005) have been found in chronic AF patients. In a previous experimental study, it has been shown that overexpression of PITX2c (a gain-of-function) increased *I*_*Ks*_ density and decreased *I*_*CaL*_density in atrial myocytes from chronic AF patients (Pérez-Hernández et al., 2015). These changes could contribute to the long-term stabilization of the arrhythmia by shortening the AP duration (APD) (González de la Fuente et al., 2012). By contrast, whilst the gain-of-function arising from the p.Met207Val mutation has been suggested to increase susceptibility to familial AF (Mechakra et al., 2019), this link remains to be demonstrated directly.

The mechanisms by which ionic and structural remodeling induced by the PITX2c p.Met207Val mutation promotes and perpetuates AF have not yet been elucidated. Complex electrical wave dynamics observed during AF is determined by AP morphology, APD, conduction velocity (CV) restitution, wavelength (WL), vulnerable window (VW) for unidirectional conduction block, and the minimal substrate size required to induce re-entry (Bai et al., 2016b; Ni et al., 2017; Whittaker et al., 2017, 2018a,b). Therefore, utilizing a multi-scale computational model of the human atria based on experimental data on the PITX2c p.Met207Val mutation, we simulated electrical activity to quantify its potential impact at the cellular, 1D fiber tissue and 2D sheet tissue levels.

## Methods

### Human Atrial Action Potential Model

**Figure 1:**
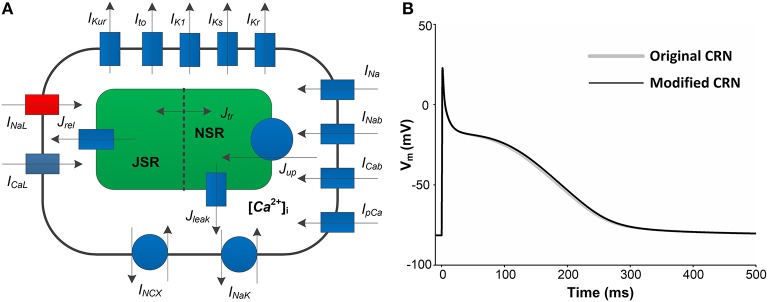
A schematic overview of human atrial myocyte model and its corresponding action potentials (APs). **(A)** The original myocyte model (Original CRN) was improved to include the *I*_*NaL*_model (marked in red). **(B)** APs obtained from the Original CRN model and the Modified CRN model.

The Courtemanche-Ramirez-Nattel (CRN) model (Courtemanche et al., 1998) of the human atrial AP was chosen to investigate the proarrhythmic effects of the PITX2c p.Met207Val mutation, because this model was suggested to study spatiotemporal characteristics of atrial fibrillation at the tissue and organ levels (Seemann et al., 2006; Kharche et al., 2012). The original CRN model was modified to reflect the observed kinetic properties of the *I*_*NaL*_ current (Figure 1A) that was based on the work of Grandi et al. (2011), who developed it using experimental data from human atrial myocardium (Sossalla et al., 2010). This *I*_*NaL*_ model is given by:

(1) $$\begin{array}{rcl} I_{NaL} & = & G_{NaL}\times m^{3}\times h\times \;\left(V_{m}-E_{Na}\right) \end{array}$$

(2) $$\begin{array}{rcl} \frac{dh}{dt} & = & \frac{h_{\infty }-h}{\tau_{h}} \end{array}$$

(3) $$\begin{array}{rcl} h_{\infty } & = & \frac{1.0}{1.0+\exp\;\left(\;\left(V_{m}+91\right)/6.1\right)} \end{array}$$

(4) $$\begin{array}{rcl} \tau_{h} & = & 600\;ms \end{array}$$

(5) $$\begin{array}{rcl} \frac{dm}{dt} & = & \frac{m_{\infty }-m}{\tau_{m}} \end{array}$$

(6) $$\begin{array}{rcl} m_{\infty } & = & \frac{\alpha_{m}}{\alpha_{m}+\beta_{m}} \end{array}$$

(7) $$\begin{array}{rcl} \tau_{m} & = & \frac{1}{\alpha_{m}+\beta_{m}} \end{array}$$

(8) $$\begin{array}{rcl} \alpha_{m} & = & \frac{0.32\;\left(V_{m}+47.13\right)}{1.0-\exp\;\left(-0.1\;\left(V_{m}+47.13\right)\right)} \end{array}$$

(9) $$\begin{array}{rcl} \beta_{m} & = & 0.08\exp\;\left(-V_{m}/11\right) \end{array}$$

where *G*_*NaL*_ (0.0025 nS/pF) is the maximal conductance, *m* and *h* are two gate variables for *I*_*NaL*_, *V*_*m*_ is the membrane potential, and *E*_*Na*_ is the sodium equilibrium potential. *m*_∞_ and *h*_∞_ denote steady-state activation and steady-state inactivation, respectively. τ_*m*_/τ_*h*_ is the time constant for *m*_∞_/*h*_∞_.

Thus, the modified CRN model is described as

(10) $$\begin{array}{r} \frac{dV_{m}}{dt}=-\frac{I_{ion}+I_{stim}}{C_{m}} \end{array}$$

(11) $$\begin{array}{r} I_{ion}=I_{Na}+I_{NaL}+I_{CaL}+I_{Nab}+I_{Cab}+I_{pCa}+I_{to}+I_{Kr}\\ +I_{Ks}+I_{Kur}+I_{NCX}+I_{NaK} \end{array}$$

where *I*_*ion*_ is the sum of all ionic currents, *I*_*stim*_ (with a duration of 0.5 ms and a strength of −80 pA/pF) is the external stimulus current and *C*_*m*_ (100 pF) is the total membrane capacitance. Compared with the original CRN model, the amplitude, duration, and shape of the AP obtained from the modified CRN model had no significant changes (Figure 1B). All the equations, parameter values and initial conditions necessary to carry out the single cell simulations in this study can be found in the Supplementary Table 1.

The modified CRN model was used to investigate the characteristics of APs. These AP features included resting membrane potential (RMP), AP amplitude (APA), the maximum depolarization rate (dV/dt_max_), AP duration at 50% repolarization (APD-_50_), AP duration at 90% repolarization (APD-_90_) and APD restitution (APDR). APDR was measured by using the standard dynamic method. The human atrial myocyte was firstly paced at a basic cycle length (BCL) of 1,000 ms for 700 beats to achieve steady-state APD_90_s and then the BCL was progressively reduced by 5 to 50 ms. APDR curves were generated by plotting APD_90_ vs. diastolic interval (DI) which was computed as BCL minus APD_90._

### Modeling Electrical and Structural Remodeling Due to the PITX2c p.Met207Val Mutation

To obtain human atrial myocyte models that reproduced the experimentally observed changes in the mRNA levels corresponding to key proteins under wild-type (WT), heterozygous (Mutant/Wild type PITX2c, MT/WT) and homozygous (Mutant PITX2c, MT) conditions (Mechakra et al., 2019), we assumed that these changes in mRNA expression are quantitatively reflected at the final functional level of ion channels and connexins. Therefore, we changed the maximal conductances of *I*_*NaL*_, *I*_*Ks*_ and *I*_*Kr*_ to account for ionic remodeling due to the PITX2c p.Met207Val mutation, and altered the diffusion coefficient (*D*) to simulate the effects of changed intercellular electrical coupling via gap junctions (Cx43 and Cx40). According to the work of Kanagaratnam et al. (2002), the Cx43/[Cx40+Cx43] ratio is associated with intercellular electrical coupling and conduction velocity (CV), such that, as the proportion of Cx40 signal increased (and that for Cx43 decreased), the CV decreased. Therefore, the Cx43/[Cx40+Cx43] ratio was used to change the parameter *D*. In our study, two different cases (i.e., MT/WT and MT) were considered for PITX2c p.Met207Val mutation-induced changes in *I*_*NaL*_, *I*_*Ks*_, *I*_*Kr*_, and *D* (Table 1).

**Table 1:** The PITX2c p.Met207Val mutation-induced changes (%) in structural and electrical components.

	**Wild-type (WT)**	**Heterozygous expression (MT/WT)**	**Homozygous expression (MT)**
*I_*NaL*_*(%)	100	190	**170**
*I_*Ks*_*(%)	100	100	**180**
*I_*Kr*_*(%)	100	260	**360**
*D* (%)	100	80	**76**

*The reduction rate of the diffusive coefficient was determined by the Cx43/[Cx43+Cx40] ratio. Under the WT condition, the Cx43: Cx40 ratio is 1:1, so the Cx43/[Cx43+Cx40] ratio is 0.5 and D (%) is set to be 100%. According to experimental data on mRNA levels of Cx43 and Cx40, D (%) is set to be 80% and 76%, respectively, under MT/WT and MT conditions. Bold values represent parameters and characteristics under the p.Met207Val PITX2c mutation condition*.

### Multicellular Atrial Tissue Models

These developed human atrial myocyte models were incorporated into 1D and 2D multicellular atrial tissue models with the modification to *D* for representing structural remodeling. These multicellular models were described by the following partial differential equation

(12) $$\begin{array}{r} C_{m}\frac{\partial V_{m}}{\partial t}=-I_{ion}+\nabla D\nabla V_{m} \end{array}$$

where *D* is a tensor describing the conductivity of the tissue and ∇ is a gradient or Laplacian operator. In one dimension, *D* = 0.031 mm^2^/ms; in two dimensions, *D*_*ij*_ = 0.031 mm^2^/ms for *i* = *j*, and *D*_*ij*_ = 0.0 mm^2^/ms for *i*≠*j*. The surface to volume ratio is 1 and *C*_*m*_ is 100 pF. These values, in the WT case, which can lead to a planar wave with a CV of 0.269 mm/ms. Time (*t*) and space (*x*) steps were set to be 0.005 ms and 0.1 mm, respectively.

In the present study, we designed three idealized geometries (a 1D cable model with 375 nodes, a 2D sheet model with 375 × 375 nodes and a homogeneous 2D tissue model with 750 × 750 nodes). In these models, the 1D cable was used to generate CV restitution (CVR) curves, and to measure CV, WL, and VW. CVR was measured by using the standard dynamic method. The 1D cable was paced at a BCL of 1,000 ms for 50 beats to achieve steady-state AP wavefronts and then the BCL was progressively reduced by 50 ms. CVR curves were constructed by plotting CV against BCL. WL was computed as the product of CV and effective refractory period (ERP). ERP and VW were calculated by using the extra stimulus method. Propagating AP wavefronts were evoked by an S1 stimulus at a BCL of 1,000 ms for 50 beats and then a test S2 stimulus applied during the refractory tail of the 50^th^ AP wave after a time delay (S1-S2 time interval). The maximal S1-S2 interval to fail to excite AP waves (bidirectional block) was defined as ERP in atrial tissues. The time window between the maximal S1-S2 interval for bidirectional block and the minimal S1-S2 interval for bidirectional conduction was defined as VW to induce unidirectional conduction block.

In addition to the temporal vulnerability of atrial tissues quantified by VW, the spatial vulnerability was evaluated in the homogeneous 2D tissue model with 750 × 750 nodes using an S1-S2 protocol. A planar wave evoked by an S1 stimulus propagated from the left side to the right side. Once this wave had passed over the first half of the domain, an S2 stimulus was applied to a center region with the same width (1.0 mm) and different lengths. The minimal substrate size was defined as the minimal size of the S2 stimulus necessary to form and sustain reentry.

To further investigate the temporal-spatial characteristics of electrical waves due to the PITX2c p.Met207Val mutation, we used the standard S1-S2 protocol to induce spiral waves in the homogeneous 2D tissue model with 375 × 375 nodes. A planar S1 stimulus was applied to the left boundary to initiate a planar wave. During the VW, a rectangular S2 stimulus was applied to the top left quarter of the domain to initiate a spiral wave. The life span of the spiral wave was measured as the time duration from initiation to dissipation. The tip meander path was traced with the method of Fenton and Karma (Fenton and Karma, 1998). Dominant frequencies of action potential (AP) profiles obtained from the central point of the domain were computed using the Fast Fourier Transform technique (Bai et al., 2016a, 2017a,b).

## Results

### Effects of the PITX2c p.Met207Val Mutation on the Human Atrial Action Potential

**Figure 2:**
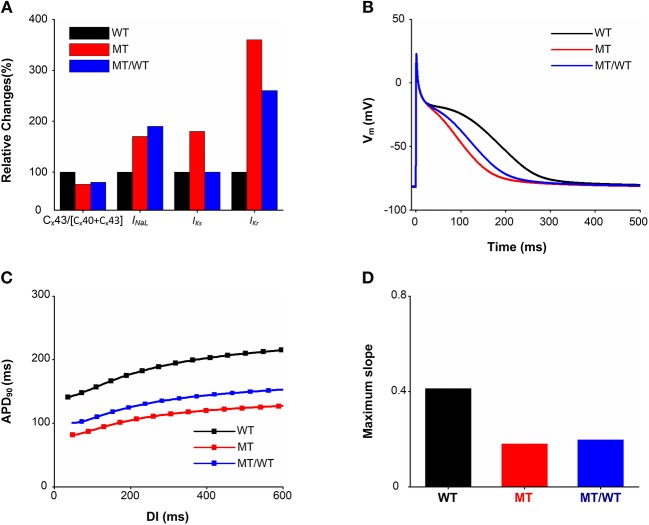
Effects of the p.Met207Val PITX2c mutation on atrial electrophysiological properties at the cellular and subcellular levels. There are three conditions (Wild type: WT, homozygous p.Met207Val mutation: MT and heterozygous p.Met207Val mutation: MT/WT). **(A)** Relative changes (%) in the Cx43/[Cx40+Cx43] ratio, *I*_*NaL*_, *I*_*Ks*_, and *I*_*Kr*_. **(B)** AP profiles. **(C)** APD restitution curves. **(D)** Measured maximum slopes of APD restitution curves.

Based on the experimental study of Mechakra et al. (2019), simulations of the functional impact of an increased transactivation activity of PITX2c due to the p.Met207Val mutation on Cx43, Cx40, *I*_*NaL*_, *I*_*Ks*_, and *I*_*Kr*_ were conducted to investigate how they contribute to atrial electrical and structural abnormalities. Figure 2A shows the relative changes caused by the gain-of-function mutation p.Met207Val (MT) include an increase in *I*_*NaL*_(1.7-fold), *I*_*Ks*_ (1.8-fold), and *I*_*Kr*_ (3.6-fold) and a reduction in the Cx43/[Cx40+Cx43] ratio (0.76-fold).

This ionic remodeling (*I*_*NaL*_, *I*_*Ks*_, and *I*_*Kr*_) arising from the PITX2c p.Met207Val mutation abbreviated human atrial APD_90_ as shown in Figure 2B. The measured APD_90_ was 260.62 ms for the WT condition, which was shortened to 197.58 ms for the MT/WT condition and to 170.14 ms under the MT condition. APA, dV/dt_max_ and RMP under the MT condition showed no significant changes as compared to the WT condition. The most obvious change of AP due to the electrical remodeling induced by the mutation was the APD shortening.

The APD shortening in the MT/WT and MT conditions was also rate-dependent as shown in Figure 2C. Across a range of DIs, the measured APD_90_s under MT/WT and MT conditions were smaller compared to the WT condition. APD restitution curves plotted by APD_90_ vs. DI were downshifted and flattened by the PITX2c p.Met207Val mutation. The computed maximal slope (0.41) for the WT condition was reduced to 0.20 and 0.18 under MT/WT and MT conditions, respectively (Figure 2D). APD shortening and flattened APD restitution curve implied that the PITX2c p.Met207Val mutation enabled human atrial cells to support electrical activity to persist at high rates.

### Effects of the PITX2c p.Met207Val Mutation on Electrical Conduction at the 1D Tissue Level

**Figure 3:**
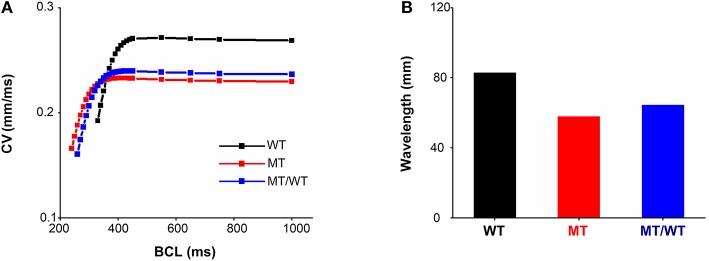
Effects of the p.Met207Val PITX2c mutation on electrical conduction at the one-dimensional tissue level. **(A)** Conduction velocity (CV) restitution curves for WT, MT, and MT/WT conditions, respectively. **(B)** Measured wavelengths at a basic cycle length (BCL) of 1,000 ms in the three cases.

In addition to ionic remodeling, structural remodeling associated with reduced intercellular coupling due to the PITX2c p.Met207Val mutation was incorporated into atrial tissue models. Using a 1D cable model, we computed CVR and WL under the WT, WT/MT, and MT conditions (Figure 3). At a BCL of 1,000 ms, the measured CV was decreased from 0.269 mm/ms for the WT condition to 0.237 and 0.230 mm/ms, under the WT/MT and MT conditions, respectively. The PITX2c p.Met207Val mutation downshifted CVR curves (Figure 3A) and facilitated electrical conduction at higher rates compared to the WT condition. The computed maximum rate of an electrical wave was increased from 194 beats/min for the WT condition to 220 and 238 beats/min, under MT/WT and MT conditions, respectively. WL abbreviation was also observed under mutant conditions (Figure 3B). The measured WL at a BCL of 1,000 ms was 82.72, 64.11, and 57.73 mm, for WT, MT/WT and MT conditions, respectively. Changes in CV and WL implied that the PITX2c p.Met207Val mutation allowed electrical waves to maintain in smaller tissue sizes that could occur under the WT condition.

### Effects of the PITX2c p.Met207Val Mutation on Temporal and Spatial Vulnerabilities of Atrial Tissue

**Figure 4:**
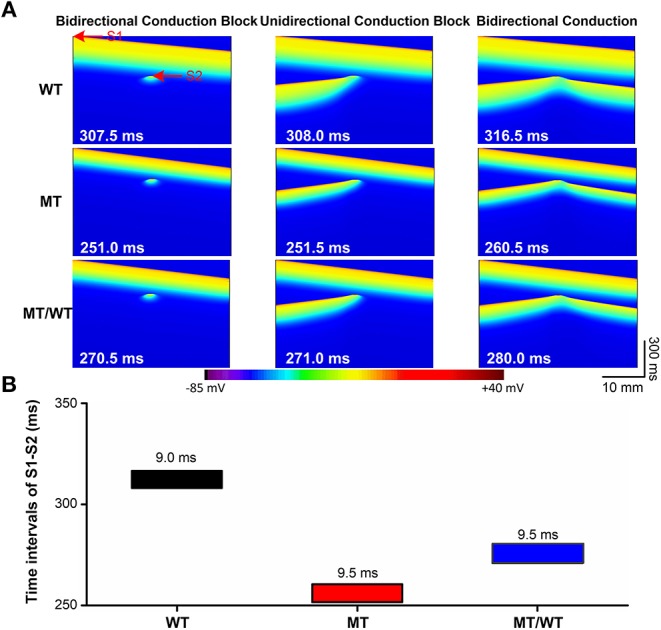
Effects of the p.Met207Val PITX2c mutation on the temporal vulnerability of atrial tissue in the 1D cable model with 375 nodes. **(A)** Electrical waves at different S1-S2 intervals, illustrating bidirectional conduction block (left), unidirectional conduction block (middle) and bidirectional conduction (right) for WT, MT, and MT/WT conditions, respectively. **(B)** Vulnerable windows of atrial tissue vulnerability to unidirectional conduction block under WT, MT, and MT/WT conditions, respectively.

The PITX2c p.Met207Val mutation led to an increase in the atrial tissue's temporal vulnerability to unidirectional conduction block indexed by VW as shown in Figure 4. Under the WT condition, the maximal S1-S2 interval for the generation of bidirectional conduction block was 307.5 ms and the minimal S1-S2 interval to induce bidirectional conduction was 316.5 ms. When S1-S2 time interval (e.g., 308.0 ms) was between 307.5 and 316.5 ms, unidirectional conduction block was induced (Figure 4A, top panel). The PITX2c p.Met207Val mutation decreased the S1-S2 time intervals, for bidirectional conduction block and bidirectional conduction, respectively (Figure 4A, middle and bottom panels). However, the width of VW was increased by the mutation (Figure 4B). The measured VW for the WT condition was 9.0 ms, which was increased to 9.5 ms for MT/WT and MT conditions.

**Figure 5:**
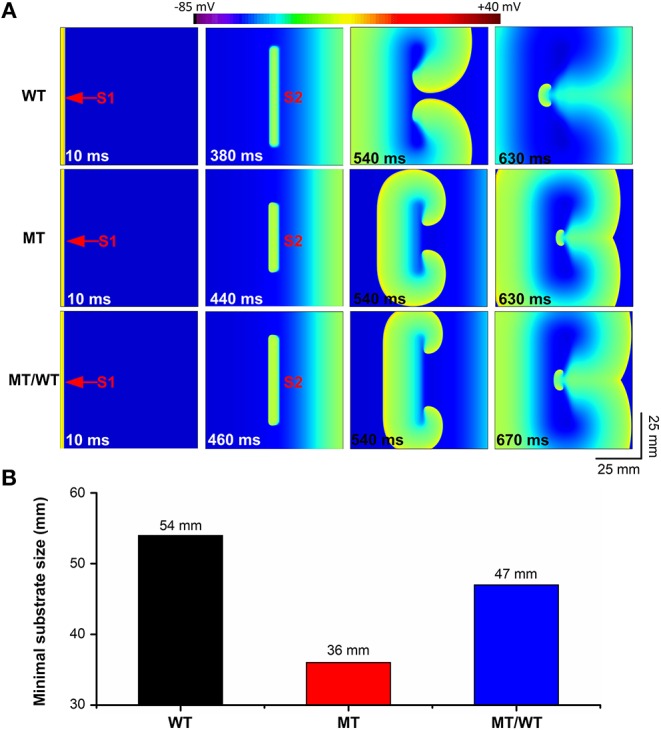
Effects of the p.Met207Val PITX2c mutation on the spatial vulnerability of atrial tissue in the 2D tissue model with 750 × 750 nodes. **(A)** Electrical waves following an S2 stimulus at different time intervals, illustrating the minimal substrate size required to induce a pair of re-entrant circuits under WT, MT, and MT/WT conditions, respectively. **(B)** Minimal substrate sizes of atrial tissue vulnerability to the genesis of re-entrant excitation waves under WT, MT, and MT/WT conditions, respectively.

The PITX2c p.Met207Val mutation caused an increase in the atrial tissue's spatial vulnerability to spiral wave formation. As shown in Figure 5A (top panel), under the WT condition, a planar wave was initiated by an S1 stimulus (time = 10 ms) and an S2 stimulus (time = 380 ms) with the minimal substrate size of 54 mm could result in sustaining reentry (time = 630 ms). The minimal substrate size was significantly decreased by the mutation from 54 to 47 and 36 mm, under MT/WT and MT conditions, respectively (Figure 5B).

Changes in VW and the minimal substrate size implied that the PITX2c p.Met207Val mutation may increase the likelihood of reentry formation.

### Effects of the PITX2c p.Met207Val Mutation on Spiral Wave Re-entry

**Figure 6:**
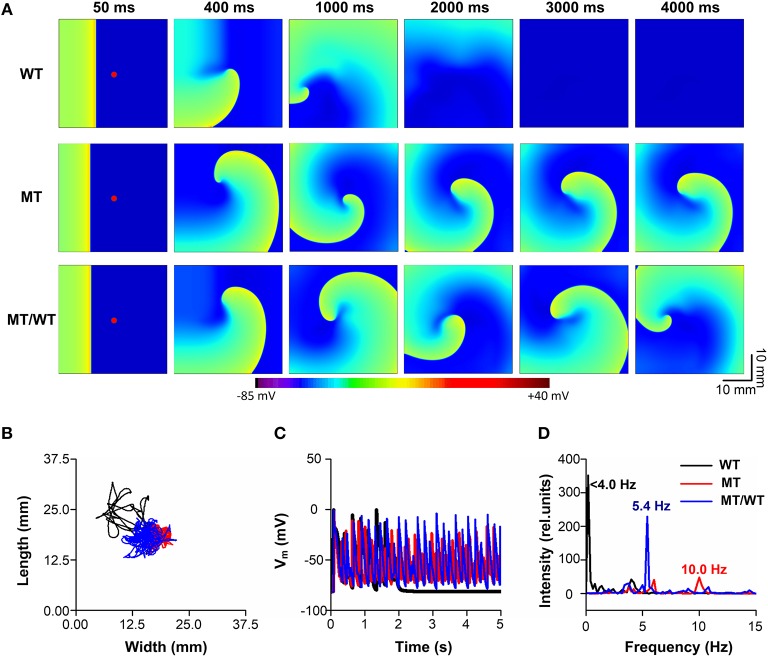
Effects of the p.Met207Val PITX2c mutation on the maintenance of spiral waves in the 2D sheet model with 375 × 375 nodes. **(A)** Snapshots of electrical waves, for WT, MT, and MT/WT conditions, at time = 50, 400, 1,000, 2,000, 3,000, and 4,000 ms. **(B)** Tip trajectories of re-entrant waves under WT, MT, and MT/WT conditions. **(C)** Membrane potential traces of localized electrical excitations (marked with red points in **(A)**. **(D)** Dominant frequencies under WT, MT, and MT/WT conditions.

To further examine whether the PITX2c p.Met207Val mutation promotes and perpetuates familial AF, its effects on the temporal-spatial dynamics of spiral waves were investigated. Figure 6A shows that the mutation led to the slow propagation of planar waves (time = 50 ms) and facilitated stable rotation (time = 4,000 ms) compared with the WT condition. The life span of the spiral wave was <2,000, >5,000 and >5,000 ms, under WT, MT/WT, and MT conditions, respectively. The meander area of the re-entrant wave trajectory under the MT condition was smaller than in the WT condition (Figure 6B). And the dominant oscillatory frequency of the membrane potential oscillations (Figure 6C) obtained from the marked point (Figure 6A, left panel) was increased from <4.0 Hz for the WT condition to 5.4 and 10.0 Hz, under the MT/WT and MT conditions, respectively (Figure 6D). Changes in life span, trajectory and dominant frequency of spiral waves implied that the PITX2c p.Met207Val mutation may increase the likelihood of familial AF (see Supplementary Videos 1–3 for details).

A quantitative summary of the proarrhythmic effects of the PITX2c p.Met207Val mutation on human atrial electrical activity is listed in Table 2.

**Table 2:** A quantitative summary of the proarrhythmic effects of the PITX2c p.Met207Val mutation on human atrial electrical activity.

**Model**	**Quantity**	**WT**	**MT/WT**	**MT**
Cell	RMP (mV)	−81.49	−81.84	–**81.98**
	APA (mV)	104.23	104.39	**104.46**
	dV/dt_max_ (mV/ms)	199.92	199.26	**199.07**
	APD_50_ (ms)	123.36	77.61	**61.26**
	APD_90_ (ms)	260.62	197.58	**170.14**
	APD restitution slope	0.41	0.20	**0.18**
1D	CV (mm/ms)	0.269	0.237	**0.230**
	ERP (ms)	307.5	270.5	**251.0**
	WL (mm)	82.72	64.11	**57.73**
	Maximum rate (beats/min)	194	220	238
	VW (ms)	8.5	9.0	**9.0**
2D	Life span (ms)	<2,000	>5,000	**>5,000**
	Dominant frequency (Hz)	<4.0	5.4	**10.0**
	Minimal substrate size (mm)	54	47	**36**
	Tip meander area (cm^2^)	2.904	1.198	**0.469**

*Bold values represent parameters and characteristics under the p.Met207Val PITX2c mutation condition*.

### Action Potential Simulations With an Alternative Human Atrial Cell Model

**Figure 7:**
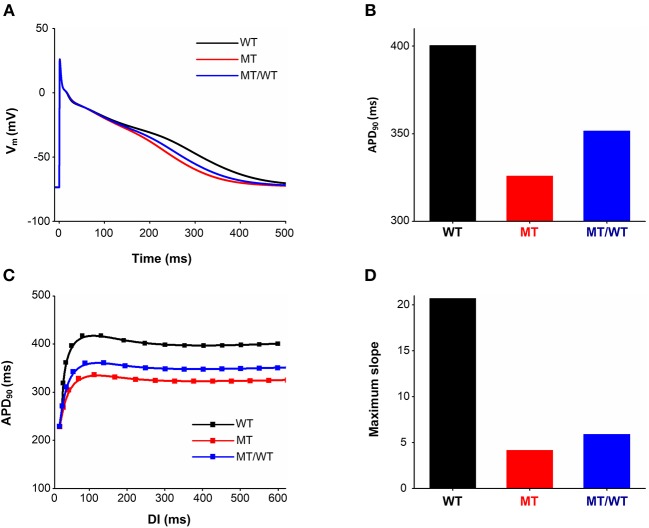
Effects of the p.Met207Val PITX2c mutation on action potential shape and action potential duration (APD) obtained from the Grandi model. AP profiles **(A)**, APDs **(B)**, APD restitution curves **(C)**, and measured maximum slopes **(D)** of APD restitution curves, for WT, MT, and MT/WT conditions, respectively.

To avoid model dependence of simulation results, we performed AP simulations using the Grandi et al. (Grand) model (Grandi et al., 2011). The abbreviated APD (Figures 7A,B) and the flattened APD restitution curve (Figure 7C) under the MT condition were observed. This mutation-induced changes in AP obtained from the Grandi model was qualitatively similar to that from the modified CRN model. APA, dV/dt_max_ and RMP under the MT condition showed no significant changes as compared to the WT condition (Table 3). In addition, the maximal slope of the APD restitution curve was reduced from 20.70 under the WT condition to 5.90 and 4.16, under MT/WT and MT conditions, respectively (Figure 7D).

**Table 3:** Quantitative characteristics of action potentials obtained from the Grandi model.

**Quantity**	**WT**	**MT/WT**	**MT**
RMP (mV)	−73.37	−73.50	–**73.58**
APA (mV)	99.19	99.62	**99.83**
dV/dt_max_ (mV/ms)	205.42	207.53	**208.56**
APD_50_ (ms)	136.04	127.44	**122.04**
APD_90_ (ms)	400.46	351.61	**325.97**

*Bold values represent parameters and characteristics under the p.Met207Val PITX2c mutation condition*.

## Discussion

### Main Findings

To our knowledge, this is the first study to investigate mechanisms underlying the generation and maintenance of re-entrant arrhythmias arising from the PITX2c p.Met207Val mutation (Mechakra et al., 2019). Our major findings are as follows: (1) This mutation-induced electrical remodeling abbreviated APD and flattened APDR curves. (2) The combined effects of electrical and structural remodeling due to the PITX2c p.Met207Val mutation slowed CV and shortened WL. (3) It increased atrial tissue vulnerability to initiation and maintenance of reentry by decreasing the substrate size to induce the figure-of-eight reentry and by increasing VW for unidirectional conduction block. (4) It allowed electrical waves to maintain in smaller tissue sizes at higher rates. Consequently, these findings demonstrate that the PITX2c p.Met207Val mutation may increase the likelihood of familial AF due to increased atrial tissue's temporal and spatial vulnerabilities, which facilitate genesis and maintenance of re-entrant excitation waves.

### Mechanisms of Familial Atrial Fibrillation Due to the PITX2c p.Met207Val Mutation

The increase of PITX2c expression or PITX2c activity has been linked to AF, as seen in the left atrium (Gore-Panter et al., 2014) and right atrial myocytes (Pérez-Hernández et al., 2015) from AF patients. In the case of the PITX2c p.Met207Val mutation, this variant was identified in 1 of 60 French patients with early-onset AF and its transactivation activity was increased 3.1-fold (Mechakra et al., 2019). In turn, this gain-of-function caused electrical and structural remodeling by modulating the mRNA levels corresponding to key proteins in AF (Mechakra et al., 2019). Although the causal link between atrial remodeling and the increased risk of AF has been demonstrated in previous modeling studies (Grandi et al., 2011; Colman et al., 2013; Koivumäki et al., 2014), the mechanisms underlying familial AF associated with the PITX2c p.Met207Val mutation were not addressed directly.

In the present study, the genesis of AF in patients with the PITX2c p.Met207Val mutation may be attributable to APD shortening due to electrical remodeling and slow conduction resulted from structural remodeling. Electrical remodeling in our models included up-regulation in *I*_*NaL*_, *I*_*Ks*_, and *I*_*Kr*_. Although upregulated *I*_*NaL*_ led to APD prolongation (Sossalla et al., 2010), the increase in *I*_*Ks*_ and *I*_*Kr*_ was found to play an important role in APD shortening (Kanaporis et al., 2019), resulting in an abbreviated WL of excitation waves that facilitated the initiation and maintenance of re-entry. Structural remodeling was modeled by decreasing the diffusion coefficient to simulate the reduced Cx43/(Cx43+Cx40) ratio. The reduction in the Cx43/(Cx43+Cx40) ratio caused slow conduction (Kanagaratnam et al., 2002; Beauchamp et al., 2006), leading to WL shortening and excitation waves at higher rates. The combined impact of electrical and structural remodeling on initiation and maintenance of atrial fibrillation can be characterized by atrial tissue's vulnerabilities to re-entry.

Effects of the PITX2c p.Met207Val mutation on the temporal and spatial vulnerabilities of atrial tissue contributed to the increased susceptibility to familial AF. According to the leading circle concept (Allessie et al., 1977), functional re-entry pathways during AF naturally assume a path length equal to the minimum circuit size for re-entry, quantified mathematically as the wavelength (or product of refractoriness and conduction velocity) (Wiener, 1946). The wavelength is therefore expected to indicate average functional re-entry circuit size. Since the maintenance of AF depends on the presence of a number of simultaneous reentering waves, and the minimum size of a reentrant wave is related to the wavelength, the wavelength should be an important determinant of the occurrence of AF. The spatial vulnerability of the atrial tissue (or the minimum length of the functional pathway necessary to sustain re-entry) was measured reciprocally by the minimal substrates to sustain re-entry (Kharche et al., 2012). In our modeling study, the formation of the induced reentrant excitation wave was dependent on the spatial size of the premature stimulus. We therefore measured the minimal size of the premature stimulus that enabled the formation of re-entry, as this is correlated with the wavelength of excitation (Kharche et al., 2012), and measures the minimal size of atrial substrate necessary to sustain re-entry. In our simulations, the PITX2c p.Met207Val mutation increased the spatial vulnerability of atrial tissue by decreasing the substrate size and WL due to abbreviated ERP and reduced CV. On the other hand, the temporal vulnerability of atrial tissue is measured as VW during which unidirectional conduction block and re-entry can be induced by a test stimulus (Zhang et al., 2008; Kharche et al., 2012). In this study, the VW was significantly increased for atrial tissue incorporating remodeling due to the PITX2c p.Met207Val mutation. Consequently, the increase of temporal and spatial vulnerabilities of atrial tissues may explain why the PITX2c p.Met207Val mutation promotes initiation of re-entry.

In addition to AF initiation, the PITX2c p.Met207Val mutation promoted the maintenance of reentry by flattening the APD restitution curves. Steady-state APD is the principal determinant of the slope of the APD restitution curve. A recent study suggested factors that prolong the action potential would be expected to steepen the restitution curve (Shattock et al., 2017). In the present study, changes in *I*_*Ks*_ and *I*_*Kr*_ contribute to APD shortening and thereby flattened APD restitution curves. APD restitution has been proposed as a mechanistic determinant of the stability of re-entrant arrhythmia (Kharche et al., 2012; Shattock et al., 2017). In the case of the PITX2c p.Met207Val mutation, the maximum slope of the APD restitution curve was reduced. This led to a gradual decrease in APD alternans toward a steady state level and thereby a stable spiral wave at the tissue level. The stable spiral wave was characterized by decreased tip meander area and prolonged life span.

Therefore, the gain-of-function of the PITX2c p.Met207Val mutation promoted AF initiation and maintenance by abbreviating APD and slowing conduction.

### Relevance to Previous Studies

Dysfunction of PITX2c predisposes to AF associated with both decreased (Wang et al., 2010; Chinchilla et al., 2011; Kirchhof et al., 2011; Kolek et al., 2014; Scridon et al., 2014; Tao et al., 2014; Aguirre et al., 2015; Lozano-Velasco et al., 2015, 2017; Herraiz-Martínez et al., 2018) and increased (Gore-Panter et al., 2014; Pérez-Hernández et al., 2015) expression (or activity) of PITX2c, and both loss- (Yang et al., 2013; Yuan et al., 2013; Zhou et al., 2013; Qiu et al., 2014; Wang et al., 2014; Wei et al., 2014) and gain-of-function of PITX2c mutations (Mechakra et al., 2019). Gain-of-function of PITX2c has previously been implicated in a marked shortening of the atrial APD and refractoriness (Pérez-Hernández et al., 2015). Overexpressed PITX2c increased the transcription of KCNQ1 and KCNE1 genes encoding *I*_*Ks*_, and decreased *I*_*CaL*_ density through the atrial natriuretic peptide in human right atrial myocytes from chronic AF patients (Pérez-Hernández et al., 2015). Significantly, previous studies have reported that the *I*_*CaL*_ decrease (Van Wagoner et al., 1999) and the *I*_*Ks*_ increase (Voigt et al., 2010) critically contribute to the APD shortening (González de la Fuente et al., 2012). Recently, there has been increasing awareness of APD shortening due to malfunction of PITX2c in AF genesis (Kirchhof et al., 2011; Bai et al., 2018). Our data indicate that, in the case of the gain-of-function mutation p.Met207Val, the increase in *I*_*Ks*_and *I*_*Kr*_not only abbreviates APD, but also increases vulnerabilities of the atrial tissue to the initiation and maintenance of re-entry. Therefore, our study adds to the growing weight of evidence implicating gain-of-function of PITX2c in increased susceptibility to AF.

### Limitations

In this study, we used both CRN and Grandi models to simulate the AP of human atrial myocytes. Although these models were developed based on experimental data on human atrial myocytes and validated by their ability to reproduce APs and calcium transients, there were several limitations discussed elsewhere (Wilhelms et al., 2013; Voigt et al., 2014; Sutanto et al., 2018). Here, the limitations special to the present study are summarized. Firstly, the diffusion coefficient (*D*) was set to be 0.031 mm^2^/ms to make the tissue model fulfill the stability criterion ($D\frac{t}{x^{2}}<0.5$) (Clayton and Panfilov, 2008; Kharche et al., 2012). Although the measured CV (~27 cm/s) is very close to realistic CVs (slow, 30 to 40 cm/s; normal 60 to 75 cm/s; and fast, 150 to 200 cm/s) (Gong et al., 2007), it is less than the human atrial CV under the normal condition (Kanagaratnam et al., 2002). Secondly, the potential impact of this mutation was evaluated under the following assumptions: (1) The PITX2c p.Met207Val mutation-induced functional changes in the proteins would be proportional to their mRNAs changes, and (2) These pro-arrhythmogenic effects would appear in human atrial cells as seen in HL1 cells. Further investigations should be conducted when more functional changes due to the PITX2c p.Met207Val mutation. Finally, the intrinsic heterogeneity, realistic geometry of the human atria and fiber orientation were not considered in this study, but these factors can influence the conduction of AP and may contribute to the genesis of spiral waves. The roles of these factors in AF were investigated in previous studies (Colman et al., 2013; Hansen et al., 2016; Zhao et al., 2017) and should not influence our conclusions. In fact, omitting these factors is useful to understand the mechanisms underlying reentrant arrhythmias arising from the PITX2c p.Met207Val mutation, in that changes in APD and spiral waves can be attributed with confidence to the implemented modifications to electrical components.

## Conclusions

In this study, our findings add to the increasing weight of evidence implicating gain-of-function of PITX2c in increased susceptibility to the genesis and maintenance of familial AF, and further highlight the impact of electrical and structural remodeling in response to the PITX2c p.Met207Val mutation. In both heterozygous and homozygous forms of this mutation led to APD shortening due to ionic remodeling and slow conduction due to structural remodeling, which together increased tissue vulnerability to arrhythmogenesis. Therefore, we concluded that APD shortening and slow conduction may increase atrial susceptibility to atrial fibrillation arising from the PITX2c p.Met207Val mutation.

## Data Availability Statement

All datasets generated for this study are included in the manuscript/Supplementary Files.

## Author Contributions

JB, JZ, and HZ conceived and designed this work. JB conducted the experiments. JB, YL, AL, and HZ drafted the manuscript, interpreted the data, reviewed, revised, and approved the final version of this manuscript.

### Conflict of Interest

The authors declare that the research was conducted in the absence of any commercial or financial relationships that could be construed as a potential conflict of interest.

## Supplementary Material

Supplementary Video 1 Electrical waves under the WT condition.

Supplementary Video 2 Electrical waves under the WT/MT condition.

Supplementary Video 3 Electrical waves under the MT condition.

## Abbreviations

1D and 2D: one- and two-dimensional model
AF: Atrial fibrillation
AP: Action potential
APD: Action potential duration
APDR: APD restitution
APD_50_: APD values at 50% repolarization
APD_90_: APD values at 90% repolarization
APA: AP amplitude
BCL: Basic cycle length
CV: Conduction velocity
CVR: CV restitution
CRN model: Courtemanche–Ramirez–Nattel model of electrical action potential of human atrial cells
D: Diffusion coefficient
DI: Diastolic interval
dV/dt_max_: Maximum depolarization rate
ERP: Effective refractory period
Grandi model: Grandi model of electrical action potential of human atrial cells
*I*_*CaL*_: L-type calcium current
*I*_*NaL*_: Late sodium current
*I*_*Ks*_: The slow delayed rectifier potassium current
*I*_*Kr*_: The rapid delayed rectifier potassium current
MT: Mutant PITX2c
MT/WT: Mutant/Wild type PITX2c
PITX2: Paired like homeodomain-2
PITX2c: PITX2 c isoform
RMP: Resting membrane potential
VW: Vulnerable window
WL: Wavelength
WT: Wild-type PITX2c.
