# Supplementary material for: Proarrhythmia in the p.Met207Val PITX2c-Linked Familial Atrial Fibrillation-Insights From Modeling
Source: Front Physiol. 2019 Oct 22;10:1314. doi: 10.3389/fphys.2019.01314 (PMC6818469; doi:10.3389/fphys.2019.01314)
Supplement: Supplementary file 1 [file Table_1.docx]

**Supplementary information**

Jieyun Bai^1*^, Yaosheng Lu^1^, Andy C.Y. Lo^2^, Jichao Zhao^2^ and Henggui Zhang^3,4*^

^1^Department of Electronic Engineering, College of Information Science and Technology, Jinan University, Guangzhou, China

^2^Auckland Bioengineering Institute, The University of Auckland, Auckland, New Zealand

^3^Biological Physics Group, School of Physics & Astronomy, University of Manchester, United Kingdom

^4^Pilot National Laboratory for Marine Science and Technology, Qingdao, China

Correspondence and requests for materials should be addressed to Jieyun Bai ([bai_jieyun@126.com](mailto:baijieyun@jnu.edu.cn))

This study aimed to reveal mechanisms underlying atrial fibrillation due to the p.Met207Val PITX2c mutation using the modified Courtemanche-Ramirez-Nattel (CRN) model. The original CRN model^1^ was modified to reflect the observed kinetic properties of the *I_NaL_* current that was based on the work of Grandi et al.^2^, who developed it using experimental data from human atrial myocardium.^3^ All the equations, parameter values and initial conditions necessary to carry out the single cell simulations in this study were provided here.

**Membrane voltage:**$\boldsymbol{V}_{\boldsymbol{m}}$

| $\frac{dV_{m}}{dt}=\frac{I_{Na}+I_{NaL}+I_{K1}+I_{to}+I_{Kur}+I_{Kr}+I_{Ks}+I_{CaL}+I_{pCa}+I_{NaK}+I_{NCX}+I_{bNa}+I_{bCa}-I_{stim}}{-C_{m}}$ |
| --- |

**Equilibrium potentials:**$\boldsymbol{E}_{\boldsymbol{K}}$**,**$\boldsymbol{E}_{\boldsymbol{Ca}}$**,**$\boldsymbol{E}_{\boldsymbol{Na}}$

| $E_{K}=\frac{RT}{F}log\frac{{{[K}^{+}]}_{o}}{{{[K}^{+}]}_{i}}$  $E_{Na}=\frac{RT}{F}log\frac{{{[Na}^{+}]}_{o}}{{{[Na}^{+}]}_{i}}$  $E_{Ca}=\frac{RT}{2F}log\frac{{{[Ca}^{2+}]}_{o}}{{{[Ca}^{2+}]}_{i}}$ |
| --- |

**Na^+^ current:** $\boldsymbol{I}_{\boldsymbol{Na}}$ **and** $\boldsymbol{I}_{\boldsymbol{NaL}}$

| $I_{Na}$=$g_{Na}m^{3}hj(V_{m}-E_{Na})$  $\alpha_{m}=\left\{ \begin{aligned} 0.32\frac{V_{m}+47.13}{1-exp\left[ -0.1(V_{m}+47.13) \right]} \\ 3.2, ifV_{m}=-47.13 \end{aligned} \right.$  $\beta_{m}=0.08exp\left( -\frac{V_{m}}{11} \right)$  $\alpha_{h}=\left\{ \begin{aligned} 0.135exp\left( -\frac{V_{m}+80}{6.8} \right) \\ 0.0, ifV_{m}\geq-40 \end{aligned} \right.$  $\beta_{h}=\left\{ \begin{aligned} 3.56\exp\left( 0.079V_{m} \right)+3.1\times{10}^{5}\exp\left( 0.35V_{m} \right) \\ \left\{ 0.13\left[ 1+exp\left( -\frac{V_{m}+10.66}{11.1} \right) \right] \right\}^{-1}, ifV_{m}\geq-40 \end{aligned} \right.$  $\alpha_{j}=\left\{ \begin{aligned} \left[ -127140\exp\left( 0.2444V_{m} \right)-3.474\times{10}^{-5}\exp\left( -0.0439V_{m} \right) \right]\frac{V_{m}+37.78}{1+exp\left[ 0.311(V_{m}+79.23) \right]} \\ 0.0, ifV_{m}\geq-40 \end{aligned} \right.$  $\beta_{j}=\left\{ \begin{aligned} 0.1212\frac{exp(-0.01052V_{m})}{1+exp\left[ -0.3178(V_{m}+40.14) \right]} \\ 0.3\frac{exp(-2.53\times{10}^{-7}V_{m})}{1+exp\left[ -0.1(V_{m}+32) \right]}, ifV_{m}\geq-40 \end{aligned} \right.$  $\tau_{\emptyset}=\left( \alpha_{\emptyset}+\beta_{\emptyset} \right)^{-1}, \emptyset_{\infty}=\tau_{\emptyset}\alpha_{\emptyset} for \emptyset=m,h, j$  $I_{NaL}=g_{NaL}m^{3}hL(V_{m}-E_{Na})$  ${hL}_{\infty}=\frac{1.0}{1.0+exp((V_{m}+91)/6.1)}$  $\tau_{hL}=600 ms$ |
| --- |

**Inward rectifier K^+^ current:**$\boldsymbol{I}_{\boldsymbol{K}\boldsymbol{1}}$

| $I_{K1}=\frac{g_{K1}(V_{m}-E_{K})}{1.0+exp\left[ 0.07(V_{m}+80) \right]}$ |
| --- |

**Transient outward K^+^ current:**$\boldsymbol{I}_{\boldsymbol{to}}$

| $I_{to}=g_{to}o_{a}^{3}o_{i}(V_{m}-E_{K})$  $\alpha_{o(a)}={0.65\left[ exp\left( -\frac{V_{m}+10}{8.5} \right)+exp\left( -\frac{V_{m}-30}{59.0} \right) \right]}^{-1}$  $\beta_{o(a)}={0.65\left[ 2.5+exp\left( \frac{V_{m}+82}{17.0} \right) \right]}^{-1}$  $\tau_{o(a)}=\left[ \alpha_{o(a)}+\beta_{o(a)} \right]^{-1}/K_{Q10}$  $o_{a(\infty)}{=\left[ 1+exp\left( -\frac{V_{m}+20.47}{17.54} \right) \right]}^{-1}$  $\alpha_{o(i)}=\left[ 18.53+exp\left( \frac{V_{m}+113.7}{10.95} \right) \right]^{-1}$  $\beta_{o(i)}=\left[ 35.56+exp\left( -\frac{V_{m}+1.26}{7.44} \right) \right]^{-1}$  $\tau_{o(i)}=\left[ \alpha_{o(i)}+\beta_{o(i)} \right]^{-1}/K_{Q10}$  $o_{i(\infty)}{=\left[ 1+exp\left( \frac{V_{m}+43.1}{5.3} \right) \right]}^{-1}$ |
| --- |

**Ultrarapid delayed rectifier K^+^ current:**$\boldsymbol{I}_{\boldsymbol{Kur}}$

| $I_{Kur}=g_{Kur}u_{a}^{3}u_{i}(V_{m}-E_{K})$  $g_{Kur}=0.005+\frac{0.05}{1+exp\left( -\frac{V_{m}-15}{13} \right)}$  $\alpha_{u(a)}={0.65\left[ exp\left( -\frac{V_{m}+10}{8.5} \right)+exp\left( -\frac{V_{m}-30}{59.0} \right) \right]}^{-1}$  $\beta_{u(a)}={0.65\left[ 2.5+exp\left( \frac{V_{m}+82}{17.0} \right) \right]}^{-1}$  $\tau_{u(a)}=\left[ \alpha_{u(a)}+\beta_{u(a)} \right]^{-1}/K_{Q10}$  $u_{a(\infty)}{=\left[ 1+exp\left( -\frac{V_{m}+30.3}{9.6} \right) \right]}^{-1}$  $\alpha_{u(i)}=\left[ 21+exp\left( -\frac{V_{m}-185}{28} \right) \right]^{-1}$  $\beta_{u(i)}=exp\left( \frac{V_{m}-185}{16} \right)$  $\tau_{u(i)}=\left[ \alpha_{u(i)}+\beta_{u(i)} \right]^{-1}/K_{Q10}$  $u_{i(\infty)}{=\left[ 1+exp\left( \frac{V_{m}-99.45}{27.48} \right) \right]}^{-1}$ |
| --- |

**Rapid delayed outward rectifier K^+^ current:**$\boldsymbol{I}_{\boldsymbol{Kr}}$

| $I_{Kr}=\frac{g_{Kr}x_{r}(V_{m}-E_{K})}{1.0+exp\left[ (V_{m}+15)/22.4 \right]}$  $\alpha_{x(r)}=0.0003\frac{V_{m}+14.1}{1-exp\left( -\frac{V_{m}+14.1}{5} \right)}$  $\beta_{x(r)}=7.3898\times{10}^{-5}\frac{V_{m}-3.3328}{exp\left( \frac{V_{m}-3.3328}{5.1237} \right)-1}$  $\tau_{x(r)}=\left[ \alpha_{x(r)}+\beta_{x(r)} \right]^{-1}$  $x_{r(\infty)}{=\left[ 1+exp\left( -\frac{V_{m}+14.1}{6.5} \right) \right]}^{-1}$ |
| --- |

**Slow delayed outward rectifier K^+^ current:**$\boldsymbol{I}_{\boldsymbol{Ks}}$

| $I_{Ks}=g_{Ks}x_{s}^{2}(V_{m}-E_{K})$  $\alpha_{x(s)}=4\times{10}^{-5}\frac{V_{m}-19.9}{1-exp\left( -\frac{V_{m}-19.9}{17} \right)}$  $\beta_{x(s)}=3.5\times{10}^{-5}\frac{V_{m}-19.9}{1-exp\left( \frac{V_{m}-19.9}{9} \right)}$  $\tau_{x(s)}=\left[ \alpha_{x(s)}+\beta_{x(s)} \right]^{-1}$/2  $x_{s(\infty)}{=\left[ 1+exp\left( -\frac{V_{m}-19.9}{12.7} \right) \right]}^{-0.5}$ |
| --- |

**L-type Ca^2+^ current:**$\boldsymbol{I}_{\boldsymbol{CaL}}$

| $I_{CaL}=g_{CaL}dff_{Ca}(V_{m}-65)$  $\tau_{d}=\frac{1-exp\left( -\frac{V_{m}+10}{6.24} \right)}{0.035(V_{m}+10)\left[ 1+exp\left( -\frac{V_{m}+10}{6.24} \right) \right]}$  $d_{\infty}{=\left[ 1+exp\left( -\frac{V_{m}+10}{8} \right) \right]}^{-1}$  $\tau_{f}={9\left\{ 0.0197exp\left[ {-0.0337}^{2}{(V_{m}+10)}^{2} \right]+0.02 \right\}}^{-1}$  $f_{\infty}{=\left[ 1+exp\left( \frac{V_{m}+28}{6.9} \right) \right]}^{-1}$  $\tau_{f(Ca)}=2$  $f_{Ca(\infty)}{=\left[ 1+\frac{{{[Ca}^{2+}]}_{i}}{0.00035} \right]}^{-1}$ |
| --- |

**Na^+^-K^+^ pump current:**$\boldsymbol{I}_{\boldsymbol{NaK}}$

| $I_{NaK}=I_{NaK(max)}f_{NaK}\frac{1}{1+\left\{ K_{m,Na(i)}/{{[Na}^{+}]}_{i} \right\}^{1.5}}\frac{{{[K}^{+}]}_{o}}{{{[K}^{+}]}_{o}+K_{m,K(o)}}$  $f_{NaK}=\left[ 1+0.1245exp\left( -0.1\frac{FV_{m}}{RT} \right)+0.0365\sigma exp\left( -\frac{FV_{m}}{RT} \right) \right]^{-1}$  $\sigma=\frac{1}{7}\left[ exp\left( \frac{{{[Na}^{+}]}_{o}}{67.3} \right)-1 \right]$ |
| --- |

**Na^+^/Ca^2+^ exchanger current:**$\boldsymbol{I}_{\boldsymbol{NCX}}$

| $I_{NCX}=\frac{I_{NCX(max)}\left\{ exp\left[ \gamma FV_{m}/(RT) \right]{{[Na}^{+}]}_{i}^{3}{{[Ca}^{2+}]}_{o}-exp\left[ (\gamma-1)FV_{m}/(RT) \right]{{[Na}^{+}]}_{o}^{3}{{[Ca}^{2+}]}_{i} \right\}}{(K_{m,Na}^{3}+{{[Na}^{+}]}_{o}^{3})(K_{m,Ca}+{{[Ca}^{2+}]}_{o})(1+k_{sat}exp\left[ (\gamma-1)FV_{m}/(RT) \right]}$ |
| --- |

**Background currents:**$\boldsymbol{I}_{\boldsymbol{Nab}}$**and**$\boldsymbol{I}_{\boldsymbol{Cab}}$

| $I_{Cab}=g_{Cab}(V_{m}-E_{Ca})$  $I_{Nab}=g_{Nab}(V_{m}-E_{Na})$ |
| --- |

**Ca^2+^ pump current:**$\boldsymbol{I}_{\boldsymbol{pCa}}$

| $I_{pCa}=I_{pCa(max)}\frac{{{[Ca}^{2+}]}_{i}}{0.0005+{{[Ca}^{2+}]}_{i}}$ |
| --- |

**Ca^2+^ release current from JSR:**$\boldsymbol{J}_{\boldsymbol{rel}}$

| $J_{rel}=k_{rel}u^{2}vw({{[Ca}^{2+}]}_{rel}-{{[Ca}^{2+}]}_{i})$  $\tau_{u}=8$  $u_{\infty}{=\left[ 1+exp\left( -\frac{F_{n}-3.4175\times{10}^{-13}}{13.67\times{10}^{-16}} \right) \right]}^{-1}$  $\tau_{v}=1.91+2.09\left[ 1+exp\left( -\frac{F_{n}-3.4175\times{10}^{-13}}{13.67\times{10}^{-16}} \right) \right]^{-1}$  $v_{\infty}=1-\left[ 1+exp\left( -\frac{F_{n}-6.835\times{10}^{-14}}{13.67\times{10}^{-16}} \right) \right]^{-1}$  $\tau_{w}=6.0\frac{1-exp\left( -\frac{V_{m}-7.9}{5} \right)}{\left[ 1+0.3exp\left( -\frac{V_{m}-7.9}{5} \right) \right](V_{m}-7.9)}$  $w_{\infty}=1-\left[ 1+exp\left( -\frac{V_{m}-40}{17} \right) \right]^{-1}$  $F_{n}={10}^{-12}V_{rel}I_{rel}-\frac{5\times{10}^{-13}}{F}\left( 0.5I_{CaL}-0.2I_{NaCa} \right)$ |
| --- |

**Transfer current from NSR to JSR:**$\boldsymbol{J}_{\boldsymbol{tr}}$

| $J_{tr}=\frac{{{[Ca}^{2+}]}_{up}-{{[Ca}^{2+}]}_{rel}}{\tau_{tr}}$  $\tau_{tr}=180$ |
| --- |

**Ca^2+^ uptake current by the NSR:**$\boldsymbol{J}_{\boldsymbol{up}}$

| $J_{up}=\frac{J_{up(max)}}{1+({{K_{up}/[Ca}^{2+}]}_{i})}$ |
| --- |

**Ca^2+^ leak current by the NSR:**$\boldsymbol{J}_{\boldsymbol{leak}}$

| $J_{leak}=\frac{{{[Ca}^{2+}]}_{up}}{{{[Ca}^{2+}]}_{up(max)}}J_{up(max)}$ |
| --- |

**Ca^2+^ buffers**

| ${{[Ca}^{2+}]}_{Cmdn}={[Cmdn]}_{max}\frac{{{[Ca}^{2+}]}_{i}}{{{[Ca}^{2+}]}_{i}+K_{m,Cmdn}}$  ${{[Ca}^{2+}]}_{Trpn}={[Trpn]}_{max}\frac{{{[Ca}^{2+}]}_{i}}{{{[Ca}^{2+}]}_{i}+K_{m,Trpn}}$  ${{[Ca}^{2+}]}_{Csqn}={[Csqn]}_{max}\frac{{{[Ca}^{2+}]}_{rel}}{{{[Ca}^{2+}]}_{rel}+K_{m,Csqn}}$ |
| --- |

**Intracellular ion concentrations:**${\boldsymbol{[Ca}^{\boldsymbol{2+}}\boldsymbol{]}}_{\boldsymbol{i}}$**,**${\boldsymbol{[Na}^{\boldsymbol{+}}\boldsymbol{]}}_{\boldsymbol{i}}$**,**${\boldsymbol{[K}^{\boldsymbol{+}}\boldsymbol{]}}_{\boldsymbol{i}}$**，**${\boldsymbol{[Ca}^{\boldsymbol{2+}}\boldsymbol{]}}_{\boldsymbol{rel}}$**，**${\boldsymbol{[Ca}^{\boldsymbol{2+}}\boldsymbol{]}}_{\boldsymbol{up}}$

| $\frac{d{{[Na}^{+}]}_{i}}{dt}=-\frac{I_{Na}+I_{NaL}+I_{Nab}+3I_{NaK}+3I_{NCX}}{V_{i}F}$  $\frac{d{{[K}^{+}]}_{i}}{dt}=-\frac{I_{K1}+I_{to}+I_{Kur}+I_{Kr}+I_{Ks}-2I_{NaK}}{V_{i}F}$  $\frac{d{{[Ca}^{2+}]}_{i}}{dt}=\frac{\left( -\frac{I_{CaL}+I_{pCa}-2I_{NCX}+I_{bCa}}{2V_{i}F}+\frac{V_{rel}J_{rel}+V_{up}(J_{leak}-J_{up})}{V_{i}} \right)}{\left( 1+\frac{\left[ Trpn \right]_{max}K_{m,Trpn}}{\left( {{[Ca}^{2+}]}_{i}+K_{m,Trpn} \right)^{2}}+\frac{{[Cmdn]}_{max}K_{m,Cmdn}}{\left( {{[Ca}^{2+}]}_{i}+K_{m,Cmdn} \right)^{2}} \right)}$  $\frac{d{{[Ca}^{2+}]}_{rel}}{dt}=\frac{J_{tr}-J_{rel}}{1+\frac{{[Csqn]}_{max}K_{m,Csqn}}{\left( {{[Ca}^{2+}]}_{rel}+K_{m,Csqn} \right)^{2}}}$  $\frac{d{{[Ca}^{2+}]}_{up}}{dt}=J_{up}-J_{leak}-J_{tr}\frac{V_{rel}}{V_{up}}$ |
| --- |

**Parameter values**

| $R$=8.3143 $J\cdot K^{-1}\cdot{mol}^{-1}$ | $T$=310 $J$ |
| --- | --- |
| $F$=96.4867 $C/{mmol}^{-1}$ | $C_{m}$=100 ${pF}$ |
| $V_{cell}$=20100 ${\mu m}^{3}$ | $V_{i}$=13668${\mu m}^{3}$ |
| $V_{up}$=1109.52 ${\mu m}^{3}$ | $V_{rel}$=96.48 ${\mu m}^{3}$ |
| ${{[K}^{+}]}_{o}$=5.4 ${mM}$ | ${{[Na}^{+}]}_{o}$=140 ${mM}$ |
| ${{[Ca}^{2+}]}_{o}$=1.8 ${mM}$ | $g_{Na}$=7.8 $nS/pF$ |
| $g_{NaL}$=0.0025$nS/pF$ | $g_{K1}$=0.09 $nS/pF$ |
| $g_{to}$=0.1652 $nS/pF$ | $g_{Kr}$=0.0294 $nS/pF$ |
| $g_{Ks}$=0.129$nS/pF$ | $g_{CaL}$=0.1238$nS/pF$ |
| $g_{Cab}$=0.00113$nS/pF$ | $g_{Nab}$=0.000674$nS/pF$ |
| $I_{NaK(max)}=$0.6 $pA/pF$ | $I_{NCX(max)}=$1600$pA/pF$ |
| $I_{pCa(max)}=$0.275$pA/pF$ | $J_{up(max)}=$0.005$mM/ms$ |
| $K_{Q10}=$3 | $\gamma=$0.35 |
| $K_{m,Na(i)}=$ 10$mM$ | $K_{m,K(o)}=$1.5$mM$ |
| $K_{m,Na}$=87.5$mM$ | $K_{m,Ca}$=1.38$mM$ |
| $k_{sat}=$0.1 | $k_{rel}=$30${ms}^{-1}$ |
| $K_{up}=$0.00092 $mM$ | ${{[Ca}^{2+}]}_{up(max)}=$15$mM$ |
| ${[Cmdn]}_{max}=$0.05 $mM$ | ${[Trpn]}_{max}=$0.07$mM$ |
| ${[Csqn]}_{max}=$10$mM$ | $K_{m,Cmdn}=$0.00238$mM$ |
| $K_{m,Trpn}=$0.0005$mM$ | $K_{m,Csqn}=$0.8$mM$ |

**Initial conditions**

| $V_{m}$=-81.2 mV | $m$=0.00291 |
| --- | --- |
| $h$=0.965 | $j$=0.978 |
| $hL$=0.043738860135 | $d$=0.000137 |
| $f$=0.999837 | $f_{Ca}$=0.775 |
| $o_{a}$=0.0304 | $o_{i}$=0.999 |
| $u_{a}$=0.00496 | $u_{i}$=0.999 |
| $x_{r}$=0.0000329 | $x_{s}$=0.01869 |
| $u$=0.00 | $v$=1.00 |
| $w$=0.9992 | ${{[Ca}^{2+}]}_{i}$=0.0001013 mM/L |
| ${{[Na}^{+}]}_{i}$=11.2 mM/L | ${{[K}^{+}]}_{i}$=139 mM/L |
| ${{[Ca}^{2+}]}_{rel}=$1.49 mM/L | ${{[Ca}^{2+}]}_{up}=$1.49 mM/L |

**Properties of action potentials,** ${{\mathbf{[}\boldsymbol{Na}}^{\mathbf{+}}\mathbf{]}}_{\boldsymbol{i}}$ **and** ${{\mathbf{[}\boldsymbol{Ca}}^{\mathbf{2+}}\mathbf{]}}_{\boldsymbol{i}}$ **in the Original CRN and the Modified CRN models driven at 1Hz from their steady states.**

|  | Original CRN | Modified CRN |
| --- | --- | --- |
| RMP (mV) | -81.50 | -81.49 |
| APA (mV) | 104.30 | 104.23 |
| dV/dt_max_ (mV/ms) | 200.22 | 199.92 |
| APD_50_ (ms) | 117.42 | 123.36 |
| APD_90_ (ms) | 256.40 | 260.62 |
| ${{[Na}^{+}]}_{i}$ | 13.30 | 13.35 |
| ${{[Ca}^{2+}]}_{i}$ | 0.000114 | 0.000115 |

**The initial values of state variables in the tissue models were set to be the values at the steady state (700 s at 1 Hz stimulation) in single cells under WT, MT/WT and MT conditions.**

|  | WT | MT/WT | MT |
| --- | --- | --- | --- |
| $V_{m}$ | -81.491781 | -81.839533 | -81.977914 |
| ${{[Na}^{+}]}_{i}$ | 13.353808 | 13.676521 | 13.795061 |
| ${{[K}^{+}]}_{i}$ | 134.672116 | 134.387074 | 134.28451 |
| ${{[Ca}^{2+}]}_{i}$ | 0.000115 | 0.000108 | 0.000106 |
| $m$ | 0.002763 | 0.002609 | 0.002551 |
| $h$ | 0.967204 | 0.969614 | 0.970524 |
| $j$ | 0.979027 | 0.980698 | 0.981320 |
| $d$ | 0.000131 | 0.000126 | 0.000124 |
| $f$ | 0.950379 | 0.967668 | 0.973744 |
| $x_{s}$ | 0.018888 | 0.018470 | 0.018321 |
| $x_{r}$ | 0.000735 | 0.000437 | 0.000355 |
| $o_{a}$ | 0.029917 | 0.029347 | 0.029123 |
| $o_{i}$ | 0.999285 | 0.999330 | 0.999348 |
| $u_{a}$ | 0.004809 | 0.004639 | 0.004573 |
| $u_{i}$ | 0.991409 | 0.991948 | 0.992165 |
| $f_{Ca}$ | 0.751808 | 0.763288 | 0.768245 |
| $hL$ | 0.137465 | 0.15093 | 0.156622 |
| ${{[Ca}^{2+}]}_{rel}$ | 1.105541 | 0.965978 | 0.908732 |
| ${{[Ca}^{2+}]}_{up}$ | 1.518534 | 1.379184 | 1.320642 |
| $u$ | 0.00000 | 0.00000 | 0.00000 |
| $v$ | 1.000000 | 1.000000 | 1.000000 |
| $w$ | 0.999213 | 0.999229 | 0.999235 |

1 Courtemanche, M., Ramirez, R. J. & Nattel, S. Ionic mechanisms underlying human atrial action potential properties: insights from a mathematical model. *American Journal of Physiology-Heart and Circulatory Physiology* **275**, H301-H321 (1998).

2 Grandi, E. *et al.* Human atrial action potential and Ca2+ model: sinus rhythm and chronic atrial fibrillation. *Circulation research* **109**, 1055-1066 (2011).

3 Sossalla, S. *et al.* Altered Na+ currents in atrial fibrillation: effects of ranolazine on arrhythmias and contractility in human atrial myocardium. *Journal of the American College of Cardiology* **55**, 2330-2342 (2010).
